# Annette Leibing and Silke Schicktanz (eds): Preventing dementia?: Critical perspectives on a new paradigm of preparing for old age: Berghahn Books, New York / Oxford, 2020

**DOI:** 10.1007/s40592-021-00135-3

**Published:** 2021-09-08

**Authors:** Niklas Petersen, Julia Perry

**Affiliations:** Department of Medical Ethics and History of Medicine, University Medical Center Göttingen, Humboldtallee 36, 37073 Göttingen, Germany

## Abstract

Given the lack of effective curative treatment options and in light of a significant reconceptualization of Alzheimer’s disease, the focus of dementia research has shifted towards prevention, risk prediction, and detection in very early disease stages. In the context of these shifts, the edited volume *Preventing Dementia?: Critical Perspectives on a New Paradigm of Preparing for Old Age* (edited by Annette Leibing and Silke Schicktanz) collects critical and insightful positions on the new paradigm of dementia prevention from an interdisciplinary and international perspective. The editors introduce the overarching topic of prevention by reflecting on the optimistic framing of modifiable risk factors and their novelty in the dementia context. Leibing and Schicktanz call for a cautious reception of the findings in the Lancet report(s) and draw attention to epistemic, ethical, and socio-political issues of what the editors term the contested “new dementia” and to the effect that this might have on rethinking individual and societal perceptions of aging. The contributions of the anthology depict the social and cultural dimensions of dementia discourses and consider the ethical implications of the changing conceptions of Alzheimer’s disease as well as the shift towards early disease stages and prevention. With this, the anthology initiates a debate about the often implicit unresolved social, ethical, and political implications and preconditions of the medical understanding and handling of cognitive disorders.

Given the lack of effective curative treatment options and in light of a significant reconceptualization of Alzheimer’s disease (AD), the focus of dementia research has shifted towards prevention, risk prediction, and detection in very early disease stages. By claiming that 40% of dementia cases could be prevented if twelve risk factors were better managed, the recent *Lancet* reports sparked broad public discussions on dementia prevention (Livingston et al. 2017, 2020). Currently, dementia and cognitive decline, accordingly, are considered less a form of ‘destiny’ or inevitability, and rather avoidable conditions which can be delayed and possibly even prevented through individual lifestyle choices.

In the context of the shifts in dementia and Alzheimer’s research, the edited volume *Preventing Dementia?: Critical Perspectives on a New Paradigm of Preparing for Old Age* (edited by Annette Leibing and Silke Schicktanz) collects critical and insightful positions on the new paradigm of dementia prevention from an interdisciplinary and international perspective. The editors introduce the overarching topic of prevention by reflecting on the optimistic framing of modifiable risk factors and their novelty in the dementia context. Leibing and Schicktanz call for a cautious reception of the findings in the Lancet report(s) and draw attention to epistemic, ethical, and socio-political issues of what the editors term the contested “new dementia” and to the effect that this might have on rethinking individual and societal perceptions of aging. This new concept aligns with a counter-movement to neuroreductionist views by strengthening the idea of the brain “becoming body” (4). The contributions of the anthology depict the social and cultural dimensions of dementia discourses and consider the ethical implications of the changing conceptions of AD as well as the shift towards early disease stages and prevention.

The first part of the edited volume focuses on social and discursive practices of dementia prevention. Lara Keuck traces and questions the changing notion of AD from a historical perspective and shows how the search for intervention strategies regarding the pathological process—for “windows to act”—have driven dementia research and, specifically, have shaped conceptualizations of AD over the past century. Leibing, followed by Schicktanz, depict how the recent paradigmatic shifts in Alzheimer’s research have been transferred into cultural practices and discourses: Based on an ethnographic study in Brazil, Leibing emphasizes how the turn towards prevention and the ‘vascularization’ of AD may likely result in victim blaming and social exclusion. In order to view prevention as an opportunity, Leibing argues that dementia prevention needs to be resituated in local contexts taking into account specific population subgroups. Schicktanz examines German scientific and public discourses on dementia, discussing the ethical implications of simplified and overstated dementia risk communication in the media and popular culture, while calling not only for culture and value sensitivity in their evaluation, but also for explicit concretizations of the prevention discourse. Pointing out the improbability of individual dementia prevention through lifestyle modifications, Matthias Leanza calls attention to the social and political preconditions for the lifestyle transformation. Alessandro Blasimme shows how the new conception of AD and the focus on prevention challenge prevailing notions of the normal and the pathological, as well as healthy cognitive aging and frailty.

In the second part of the anthology, Tiago Moreira, as well as Stephen Katz, Kevin Peters, and Peri Ballantyne, describe the evolution of the disease label *Mild Cognitive Impairment* (MCI), which describes the transitional state between normal cognitive performance and cognitive ability loss, which can potentially lead to AD or a related dementia. Moreira traces the history of MCI in the last two decades, while questioning its explanatory power. First considered a specific risk factor for AD, MCI is now understood in a broader sense as cognitive decline caused by a variety of possible etiologies. Based on interviews and focus groups Katz, Peters, and Ballantyne show how the diagnostic uncertainties surrounding MCI are negotiated among researchers, practitioners, and affected persons, while drawing on the importance of collective responsibility.

The last section focuses on conceptual and normative implications of the preventive turn in dementia research. Comparing the current discourse on dementia prevention to older chronic illness discourses primarily in the US, with a specific focus on cancer, Kirsten Bell shares her astonishment that dementia is only now being negotiated as preventable through lifestyle changes. Mark Schweda and Larissa Pfaller discuss the critique of the responsibilization of cognitive aging and call for a differentiated analysis of responsibility ascriptions in the context of dementia prevention. Finally, Thomas Foth critically discusses how the logics and the role of health policy and state intervention change with the new focus on prevention and shows how the lifestyle paradigm is associated with strong normative judgment about healthy living and socially responsible self-care. In the afterword, Peter Whitehouse and Daniel George reflect on the concepts of cure and prevention and articulate the hope that the critical discussion of the conceptions and futures of AD and prevention “can serve as a gateway to asking larger questions about what kind of societies we want to have” (248).

By posing this larger question and situating the paradigmatic shifts in Alzheimer’s and dementia research within current aging cultures and contemporary social policies, the anthology initiates a debate about the often implicit unresolved social, ethical, and political implications and preconditions of the medical understanding and handling of cognitive disorders. However, further empirical analysis on specific dementia discourses and their practical implications is needed to adequately meet the claim of ‘situating’ the current understanding of dementia. The cultural-scientific and ethical discussion on the current handling of dementia and prevention would benefit from a more precise examination of specific dementia discourses in different national and scientific contexts. In addition, empirical studies are needed to examine in which ways the novel scientific understanding of dementia actually affects individual as well as societal notions of risk and responsibility.
